# Congenital Phimosis with Adherent Prepuce

**Published:** 1878-07-20

**Authors:** E. C. Lemen


### CONGENITAL PHIMOSIS WITH ADHERENT PREPUCE.

BY E. C. LEMEN, M. D.

Read before the Madison County Medical Society.

That phimosis may become a source of many inconveniences as well as contribute to the production of organic diseases, as balinitis, cystitis and irritation if not inflammation of the kidneys, has been fully dwelt on by authors and lecturers, as well as established by the observation of all regular physicians.

But the object of the present essay is to call the minds of the members of the society to a condition which I am certain often follows the above pathological conditions as a natural sequence. And which I believe might be, and doubtless has been, in many instances referred to other causes, much to the detriment of the patient, if not ultimately to chagrin of the physician. We propose to consider phimosis with adherent prepuce as a factor in the production of *spinal irritation, or spinal anœmia;* which may or may not result in reflex spasm, or reflex paralysis.

It is an universally admitted physiological fact that a peripheral irritation in any part of the body, if long continued may result in either reflex spasms or reflex paralysis. The honor of first directing the attention of the medical profession to a condition of semi-paralysis as well as the loss of the power of co-ordination of muscular action from the above causes, is due to Professor Sayers of New York City, in a clinical lecture delivered Oct. 14, 1870.

Previous to this time no author or lecturer on either diseases of the nervous system, or genito uniary organs, had so much as made reference to the above results as dependent upon, or in consequence of the pathological conditions under consideration.

The source of irritation in the case is to be found in the retention and deposit of the sebaceous excretion of the corona glands, the liquid portion being absorbed, the calcarious portion remaining as a hard ring or concretion. The result of a series of cases presented to and treated by Professor Sayre will illustrate.

In which many or all of the following symptoms and conditions were present: Extreme restlessness, wakefulness, night terrors, priapism, urinary troubles, inability to speak correctly, unsteady gait, tumbling down, loss of power of co-ordination, spasmodic action, and finally paralysis.

A synopsis of two of his cases will suffice to illustrate.

Case No. 1. Aged four years, had never walked or talked; face idiotic, convulsive movements of both upper and lower extremities, lower extremities rigid, tendo Achillis contracted as in talipes equinus; prepuce elongated and adherent. Operated and removed hard ring of smegma from corona.

A few weeks later patient talked, walked, slept well and fed himself as a child of his age usually does.

Case No. 4. History as given by patient's father. Patient three years old, very restless at night. had never slept two consecutive hours at night, and then on his hands and knees, irritable, peevish and vicious, tottering and unsteady in walking, etc.

By request of friends he had consulted Prof. Sayers, who on examination felt along the spine; and by pressure at a certain point, spasmodic movements of the child's limbs occurred. Examined prepuce, retracted foreskin. Operated by splitting foreskin and removing something which looked like a sliver of bone. Ten days after operation, patient began to eat and sleep well, and is becoming so docile that we hope in a short time he will act like a white man's child. Prof. Sayer then said "there is some discussion as to whether reflex irritation and paralysis can be produced by the above conditions ; but that a score of like cases operated on by him and recovering perfectly without further treatment, is conclusive evidence."

Drs. Beardsly and Hall have reported a number of cases in every respect similar to those of Dr. Sayer, both in course of history of cases and results of operations.

One of Dr. Beardsly's cases will suffice to illustrate.

Patient seven years old, with complete paralysis of lower extremities of two months duration ; convulsive movements preceding paralysis.

Adherent prepuce with signs of recent inflammation. Circumcised and turned out from beneath the prepuce a pent up deposit of sebaceous matter. The patient rapidly recovered without further treatment. I am able to offer three cases quite similar to the above ; in all of which there existed phimosis and adherent prepuce.

The cases all presented the following history ; capricious appetite, imperfect nutrition, irritable temper priapism , insomnia unsteady gait ; semi-choreic, or partial loss of power of co-ordination slight epileptiform convulsions but no paralysis. But doubtless had the cases been neglected and the cause of irritation remained, the next step in the pathological action would have been paralysis.

I was induced to operate by having read the results of the cases of Drs. Sayre, Beardsley and Hall, who operated upon all and in each case found phimosis and adherent prepuce together with the hard ring of smegma at corona; the results perfectly charming in each case; in a few days after operation, all of the unpleasant symptoms above enumerated vanished as would a mist before the morning sun.

Another effect of phimosis either direct or indirect; is the formation of a stone in the urethra or bladder; (and especially of the phosphatic calculli.)

I have met with four cases of stone in the bladder and one case of urethral calculus with fistula urethræ, in the past fifteen months; and in all phimosis existed.

In consequence of the contracted preputial orifice it is necessary for the patient to make use of an unusual amount of force to expell the urine.

Consequently the bladder is never entirely or completely emptied at the time of urinating. The retained urine becomes decomposed and ammoniacal, cystitis is developed, the salts of the urine—especially the phosphates—are precipitated, resulting in the formation of stone.

Dr. Packard of Philadelphia says many of the symptoms of stone in the bladder may be produced by phimosis alone.

Twice in twelve months had he operated for phimosis in which the only symptom absent was hæmaturia. All trouble disappeared after operation.

Statistics prove that in half the cases of male children the glands penis cannot be exposed, the prepuce being too long or narrow. It may be true though that in many of these cases nature is capable of removing to a very great extent the trouble before the patient attains the age of puberty. Still the fact remains to be met, that the many cases would have been benefitted by an early operative interference.

I would therefore submit the suggestion; that it is the duty of Physicians to examine male children who may be under their professional care, and operate upon the same when in their opinion it would be for the best interest of the patient.

And especially should they present urinary trouble; or mal nutrition with vague nurotic symptoms; for as is well known a slight peripheral irritation may result, if long continued, in serious functional disturbance.—*St. Louis Medical Journal.*

